# Light–Heat Interactions in Photothermal Ammonia Synthesis: A Diagnostic Framework for Mechanistic Interpretation

**DOI:** 10.1021/acsami.6c08744

**Published:** 2026-06-01

**Authors:** Javier Fermoso, Meryem Farchado, Laura Collado

**Affiliations:** † 83076 Instituto de Catálisis y Petroleoquímica (ICP), CSIC, c/Marie Curie 2, 28049 Madrid, Spain; ‡ 202532 Photoactivated Processes Unit, IMDEA Energy Institute, Parque Tecnológico de Móstoles, Avda. Ramón de la Sagra 3, Móstoles, 28935 Madrid, Spain

**Keywords:** photothermal ammonia synthesis, photothermal catalysis, nitrogen reduction reaction, mechanistic interpretation, light−heat interactions, reaction kinetics, ruthenium catalysts, iron catalysts

## Abstract

Photothermal ammonia synthesis has emerged as a promising route to couple nitrogen fixation with solar energy, yet the mechanistic origin of light-assisted activity remains difficult to establish because illumination can contribute through heat generation, photoexcited carriers, or both simultaneously. This Review examines recent photothermal NH_3_ synthesis systems from a diagnostic perspective. Representative Ru- and Fe-based materials are discussed within four photothermal regimes: photo-driven thermocatalysis, photo-assisted thermocatalysis, photothermal co-catalysis, and thermally assisted photocatalysis; and are evaluated according to two complementary questions: what is the most likely dominant photothermal regime under the reported reaction conditions, and how strong is the mechanistic evidence supporting that assignment? Particular attention is given to temperature-normalized light/dark comparisons, apparent activation energy and reaction-order analysis, wavelength- and intensity-dependent activity, isotopic validation, operando spectroscopy, and methods for constraining local thermal gradients. Across the strongest cases, the clearest mechanistic effects emerge when light-responsive functionality overlaps with the kinetically relevant elementary step at a catalytically active interface. The analysis also shows that photothermal co-catalysis should be assigned more restrictively than the other regimes and only when simultaneous and cooperative thermo-photochemical contributions are supported by sufficiently robust mechanistic evidence. By comparing Ru- and Fe-based systems through this evidence-based framework, this Review identifies structure-, interface-, and kinetic-constraint-dependent trends, proposes practical criteria for mechanistic assignment, and outlines priorities for catalyst design, reporting standards, and reactor development in photothermal ammonia synthesis.

## Graphical abstract

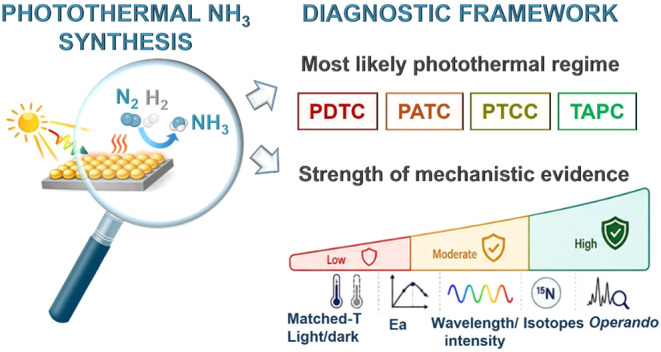

## 1 Introduction

Ammonia remains central to the emerging decarbonized energy and chemical landscape. Beyond its indispensable role in fertilizer production, it is increasingly regarded as a carbon-free hydrogen carrier and a potential energy vector. Yet industrial ammonia synthesis is still dominated by the Haber-Bosch process, which operates at elevated temperatures and pressures and involves substantial energy demand and CO_2_ emissions.^1,2^ Developing alternative routes for sustainable NH_3_ synthesis is therefore a major scientific and technological challenge, motivating intense efforts to couple nitrogen activation more directly to renewable energy inputs.^3,4^

Among the emerging approaches, photothermal ammonia synthesis occupies a particularly intriguing position because it operates at the interface between thermocatalysis and photocatalysis. In these systems, absorbed photons may be dissipated predominantly as heat, may generate photoexcited carriers, or may do both simultaneously, with the balance depending strongly on absorber identity, catalyst architecture, and reaction conditions.^5–9^ Depending on whether light is harvested by metallic nanoparticles, defective semiconductors, hybrid interfaces, or carbon-based materials, illumination may lead to localized photothermal heating, spatial temperature gradients, nonequilibrium carriers, or interfacial charge redistribution that perturbs adsorption and surface reaction energetics.^7–9^ As a result, photothermal NH_3_ synthesis cannot be interpreted solely in terms of either conventional thermocatalysis or purely light-driven nitrogen reduction. Instead, it encompasses a continuum of situations in which heat generation, carrier excitation, and heat and mass transport may all contribute to the observed catalytic response.^5,6,10^

This complexity has made mechanistic interpretation one of the central unresolved issues in the field. In many reports, improved NH_3_ productivity under illumination can be rationalized by bulk heating, local hot spots, or favorable thermal gradients. In other cases, illumination appears to modify apparent activation energies, reaction orders, catalyst inhibition patterns, or the identity of kinetically relevant elementary steps. However, because optical absorption, heat generation, and catalytic turnover occur within the same irradiated reactor environment, the temperature that controls catalysis is not necessarily identical to the temperature measured macroscopically. Consequently, activity enhancement under illumination does not by itself demonstrate intrinsic photoinduced modification of the reaction pathway.^5,7–10^ This distinction is especially important in ammonia synthesis, where modest temperature bias can strongly affect apparent rates, equilibria, and inferred kinetic parameters. Recent analyses of gas–solid photothermal catalysis have further shown that unresolved spatial gradients and inappropriate thermometry can lead to substantial temperature bias, making rigorous thermal normalization a prerequisite for any mechanistic interpretation of light–heat synergy.^11,12^

Several recent reviews have summarized the fundamentals of photothermal catalysis, broader light-driven nitrogen fixation strategies, and the opportunities and challenges of photo­(thermal) NH_3_ synthesis from catalyst and process perspectives.^4–6,10^ More recently, dedicated reviews have also appeared on photothermal catalytic ammonia synthesis and on light-driven NH_3_ synthesis from N_2_ and H_2_, further expanding the field-level overview of catalyst classes, reaction concepts, and mechanistic possibilities.^13,14^ What remains less resolved, however, is how to evaluate the quality of evidence behind mechanistic claims in reported photothermal ammonia synthesis systems. More specifically, which combinations of experiments are sufficient to distinguish predominantly thermal acceleration, localized photothermal effects, and true photoinduced perturbation of the catalytic pathway under reaction conditions? Addressing that question requires more than listing catalyst performances or assigning systems to rigid categories; it requires a critical assessment of what each experimental observable can and cannot demonstrate.

In this Review, we therefore adopt a diagnostic perspective (Scheme 1). Rather than treating reported systems as members of fixed and mutually exclusive classes, we discuss them within four operational categories commonly used in photothermal catalysis: photo-driven thermocatalysis (PDTC), photo-assisted thermocatalysis (PATC), photothermal co-catalysis (PTCC), and thermally assisted photocatalysis (TAPC), and assess them according to two complementary questions: (i) what is the most likely dominant photothermal regime under the reported reaction conditions, and (ii) how strong is the mechanistic evidence supporting that assignment? To answer these questions, we place particular emphasis on temperature-normalized light/dark comparisons, reaction order and apparent activation energy analysis, wavelength- and intensity-dependent activity at controlled temperature, isotope-based experiments, in situ and *operando* spectroscopy, and control strategies designed to constrain local thermal gradients or separate photothermal from photoinduced contributions.^5,6,10–12^ These categories are used here as practical descriptors rather than rigid ontological classes, since thermal and photoinduced contributions often coexist under reaction conditions; among them, photothermal co-catalysis is reserved for cases in which both thermo- and photoinduced pathways appear to contribute simultaneously and cooperatively to the observed catalytic response.

**Scheme 1:**
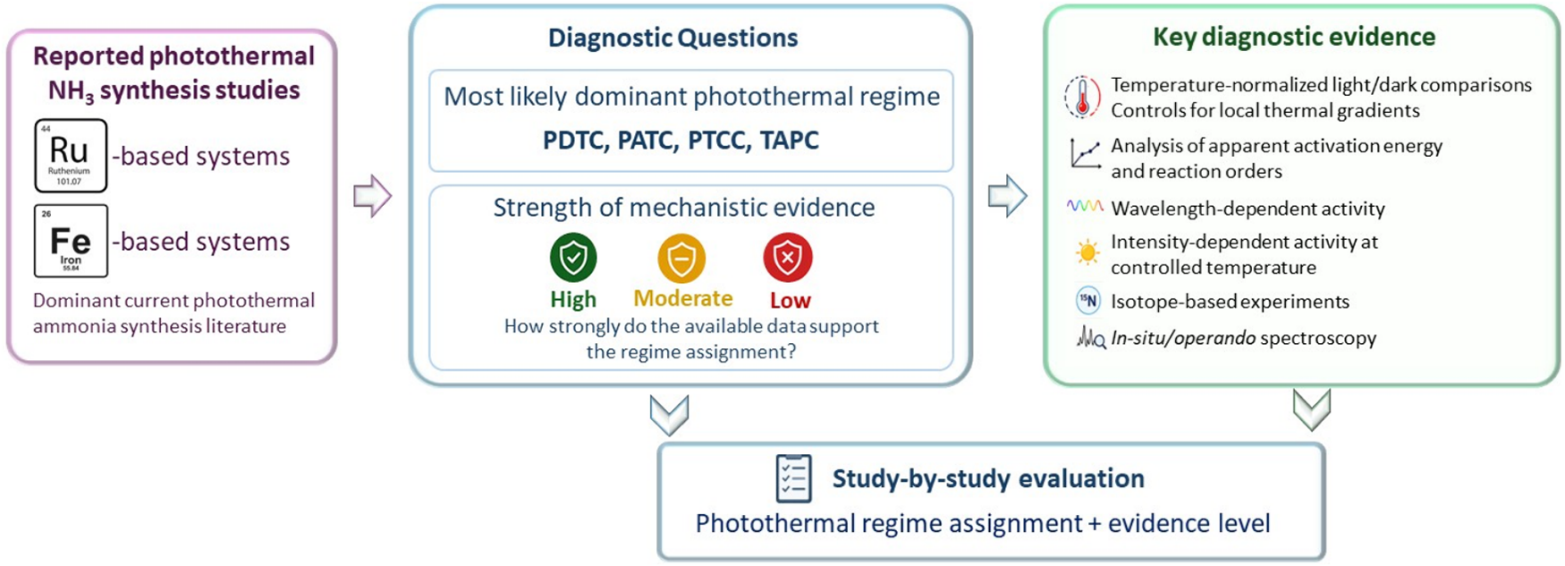
Diagnostic Framework for Assessing Photothermal NH_3_ Synthesis Studies

We apply this evidence-based framework to representative Ru- and Fe-based catalysts, which dominate the current photothermal ammonia synthesis literature because of their established relevance to thermocatalytic NH_3_ production and their contrasting kinetic constraints. Ruthenium-based systems provide the clearest cases in which illumination can measurably perturb kinetically relevant steps under intrinsically thermal reaction conditions, particularly when reducible supports and promoter effects facilitate interfacial electronic redistribution. Iron-based catalysts, in contrast, highlight the greater complexity introduced by stronger sensitivity to nitrogen dissociation barriers, ammonia inhibition, and structure-dependent active-site requirements.^4,6^ By comparing how illumination interacts with these metal- and structure-dependent kinetic landscapes, this Review aims not only to critically evaluate reported claims of photothermal synergy, but also to extract practical guidance for catalyst design, mechanistic validation, and reactor operation in solar-driven ammonia synthesis.

## 2 Diagnostic Framework for Photothermal Ammonia Synthesis

### 2.1 Classification of Photothermal Catalytic Regimes

Assigning a photothermal ammonia synthesis system to a given mechanistic regime requires more than observing enhanced NH_3_ formation under illumination. In irradiated catalytic reactions, photon absorption, nonradiative heat generation, carrier excitation, and heat and mass transport occur simultaneously, and several of the experimental signatures commonly invoked to claim photothermal synergy, such as reduced apparent activation energy, altered reaction orders, or light-intensity-dependent activity, may also be influenced by unresolved local thermal effects.^5,6,10–12,16^ A rigorous mechanistic interpretation therefore requires separating two questions that are often implicitly treated as equivalent: what is the most likely dominant photothermal regime under the reported reaction conditions, and how strong is the evidence supporting that interpretation?

To align the present Review with the broader photothermal catalysis literature,^9,10,17,18^ we retain the four operational categories commonly used to describe light–heat coupling in heterogeneous catalysis: (i) photo-assisted thermocatalysis (PATC), (ii) photo-driven thermocatalysis (PDTC), (iii) photothermal co-catalysis (PTCC), and (iv) thermally assisted photocatalysis (TAPC), as illustrated in Scheme 2. This classification is used as a conceptual framework for describing how thermochemical and photochemical contributions are coupled under irradiation, while the strength of evidence supporting assignment to any given category is assessed separately. This distinction is especially important in photothermal ammonia synthesis, where apparent light promotion may arise from bulk heating, local thermal gradients, carrier-mediated effects, or combinations thereof.

**Scheme 2:**
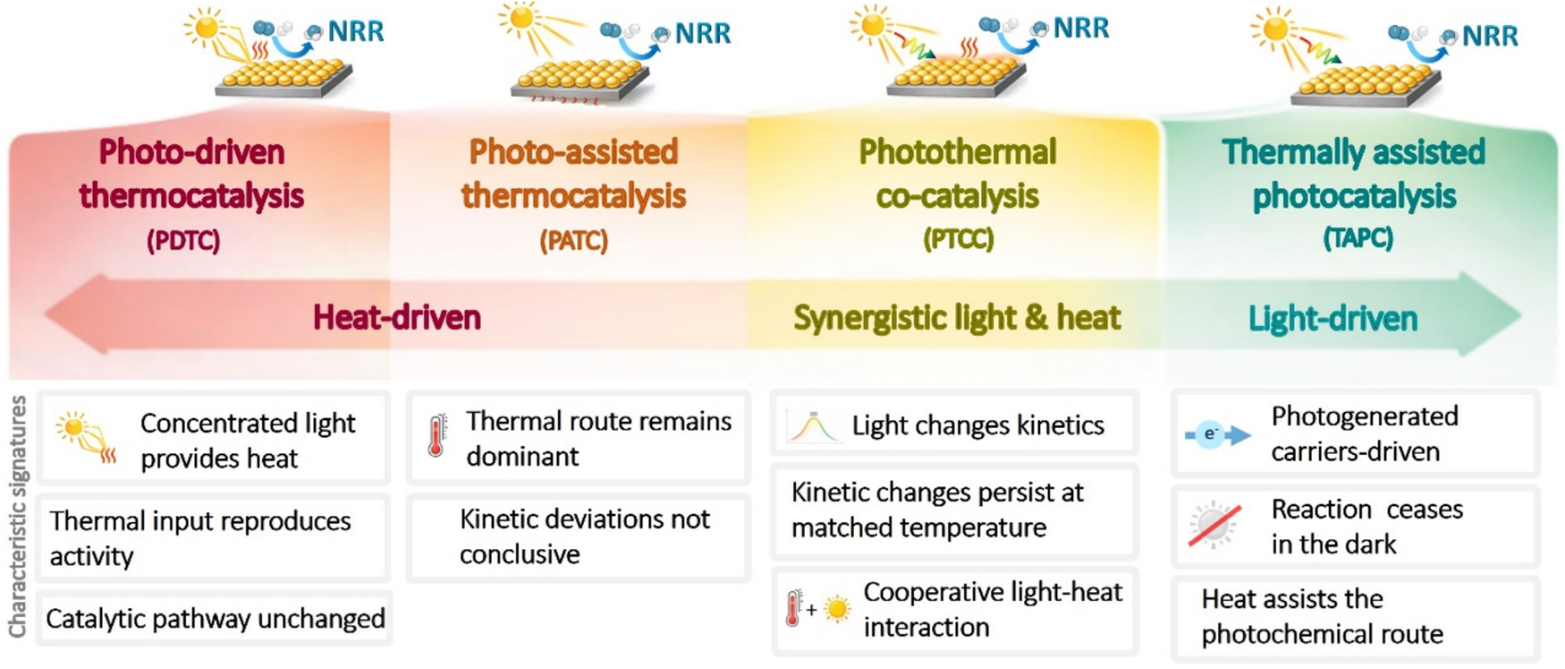
Classification of Photothermal Catalysis Regimes Based on the Dominant Driving Force in the Ammonia Synthesis^a^

a Photo-driven thermocatalysis (PDTC) and photo-assisted thermocatalysis (PATC), where activity enhancements can be fully rationalized by temperature rise or heat localization; photothermal co-catalysis (PTCC), where synergistic light-heat interactions intrinsically modify reaction energetics; and thermally assisted photocatalysis (TAPC), where the reaction is primarily driven by photoexcited charge carriers with additional thermal contributions

(i) Photo-assisted thermocatalysis (PATC) describes systems that remain fundamentally thermally activated. Photon energy is converted into heat that directly drives an otherwise conventional thermocatalytic reaction. Under isothermal comparison, the catalyst exhibits comparable activity whether heated optically or externally. Any enhancement observed under illumination can be fully rationalized by bulk or localized temperature increases, without compelling evidence that the intrinsic catalytic route differs from that under dark thermal operation.
(ii) Photo-driven thermocatalysis (PDTC) may formally be regarded as an extreme subtype of photo-assisted thermocatalysis, where concentrated solar irradiation functions as an alternative heat source, substituting conventional heating without modifying surface energetics.
(iii) Photothermal co-catalysis (PTCC) describes systems in which thermochemical and photochemical effects coexist and interact. The reaction remains thermally activated, yet illumination induces measurable intrinsic kinetic modifications under strictly comparable thermal conditions. Light does not merely increase temperature nor simply introduce an independent photocatalytic pathway; instead, it alters surface energetics, activation barriers, or dominant reaction routes in ways that cannot be reproduced by external heating alone. Reduced apparent activation energy under illumination at fixed temperature, altered reaction orders, light-intensity dependence independent of bulk heating, or *operando* evidence of photoinduced electronic redistribution at catalytic sites constitute key signatures of this regime. Assignment to this synergistic regime requires rigorous isothermal discrimination to exclude contributions arising solely from localized heating or thermal gradients. In practice, however, some systems exhibit partial or suggestive kinetic deviations under illumination without fully excluding localized photothermal effects. Thus, this category is the most demanding mechanistically, because it requires evidence not only that both pathways operate, but also that their coupling produces genuinely synergistic behavior.
(iv) Thermally assisted photocatalysis (TAPC) refers to systems in which the photochemical pathway is primary, while thermal effects play a secondary role by accelerating the overall reaction. Ammonia formation typically occurs at relatively low bulk temperatures under illumination and diminishes or ceases in the dark, even if moderate heating is maintained. The defining features of this regime include wavelength-dependent activity matching optical absorption, direct evidence for photogenerated charge-carrier formation and participation in bond activation, and *operando* spectroscopic signatures of carrier involvement. Here, thermal input mainly enhances charge-carrier mobility, mass transport, and subsequent surface reaction steps, but does not constitute the primary activation pathway, which is mediated by photogenerated charge carriers.

These categories should not be treated as perfectly discrete mechanistic classes with sharp boundaries. In real photothermal systems, thermal and photoinduced contributions often coexist, although in many cases one regime provides the best description of the dominant catalytic behavior under the reported conditions. Their relative importance may vary with catalyst composition, irradiation conditions, pressure, temperature, and reactor configuration.^16,19,20^ Accordingly, in this Review the four-category classification is used to identify the most plausible mode of light–heat coupling, while the confidence of each assignment is evaluated independently from the available temperature-normalized controls, kinetic discrimination, spectroscopic evidence, and isotopic validation. This two-dimensional approach is particularly useful for photothermal NH_3_ synthesis, where claims of true coupling or co-catalysis should be reserved for cases in which simultaneous and synergistic thermo-photochemical contributions are supported by robust mechanistic evidence rather than inferred from activity enhancement alone. Systems with suggestive but incomplete data sets are discussed as cases in which the confidence of the assignment remains limited because alternative explanations, especially local thermal artifacts, cannot yet be sufficiently excluded. This conceptual two-dimensional approach underlies the interpretive framework used throughout the following sections and should be read together with Tables 1 and 2.

**Table 1:** Diagnostic Tools for Interpreting Light–Heat Interactions in Photothermal Ammonia Synthesis^a^

diagnostic approach	primary diagnostic value	main limitation	recommended complementary evidence	refs
Isothermal light/dark kinetics	Tests whether illumination enhances NH_3_ synthesis beyond the effect of the measured catalyst temperature	Nominal temperature matching may still mask local thermal gradients	Local thermometry, Arrhenius analysis, reaction orders, optical controls	^11,12^
Kinetic signatures under illumination (apparent activation energy, reaction orders, intensity dependence at fixed temperature)	Probes whether illumination modifies the effective kinetic response, inhibition patterns, or apparent rate-controlling regime	These observables are phenomenological and may be distorted by temperature bias or mass transport effects	Isothermal controls, local temperature measurements, isotopic experiments, *operando* spectroscopy	^6,10–12,16,18^
Spectral and optical control experiments (wavelength dependence, optical filtering, direct vs indirect illumination, chopped-light response)	Tests whether catalytic behavior depends on catalyst photoexcitation rather than only on external heating	Thermal and electronic effects may both be wavelength- or light-profile-dependent	Thermal normalization, local thermometry, *operando* spectroscopy	^5,7,8,10,19,20^
Isotopic experiments (^15^N_2_ labeling/exchange)	Verifies nitrogen source and probes whether illumination affects N_2_ activation or subsequent hydrogenation steps	Does not by itself identify the dominant photoinduced contribution	Temperature-normalized kinetics, *operando* spectroscopy	^4,6,10^
*Operando* and in situ spectroscopy (DRIFTS, Raman, XPS)	Reveals illumination-induced changes in surface chemistry	Spectral changes do not automatically identify the rate-controlling step	Temperature-normalized kinetics, isotopic experiments	^4,6,10,16^
Excited-state probes (photocurrent, PL, transient absorption)	Confirms photoexcitation and charge carrier dynamics	Does not by itself demonstrate catalytic participation in kinetically relevant steps	Temperature-normalized kinetics, optical controls, *operando* spectroscopy	^4,6,10,16^
Thermometry and transport analysis (IR, pyrometry, blackbody methods, *operando* XRD, heat/mass transport modeling)	Assesses whether the measured temperature adequately represents the catalytic thermal state	No single method fully resolves active-site temperature under reaction conditions	All kinetic observables	^11,12,19–21^

a The table summarizes the principal experimental approaches used to evaluate how illumination influences catalytic performance. It highlights primary diagnostic values, main limitations when interpreted in isolation, and the complementary evidence needed for robust mechanistic understanding.

**Table 2:** Evidence-Based Comparison of Representative Photothermal Ammonia Synthesis Systems Reported Since 2018^a^

catalyst	reaction conditions^b^	NH_3_ rate^c^	matched-T light/dark control^d^	kinetic perturbation under light^e^	additional mechanistic support^f^	main residual uncertainty	likely dominant photothermal regime^g^	evidence level^h^	refs
Ru–Cs/MgO	N_2_ + H_2_, 333 °C, Blue LED (4.7 W cm^–2^)	4.5	N	N	Equivalent-temperature concept; thermal-gradient engineering	No evidence that illumination changes the intrinsic pathway	PDTC	H	^26^
CsRu@SrTiO_3_	N_2_ + H_2_, 360 °C, 300 W Xe lamp (0.1 W cm^–2^)	3.5	Y	N	NIR response; similar apparent *E*_a_ in light and dark	Hot-carrier contribution proposed, but kinetic pathway remains essentially thermal	PATC	M	^27^
Ru–Cs/ZrO_2_	N_2_ + H_2_, 350 °C, 300 W Xe lamp (0.1 W cm^–2^)	5.1	Y	Y	Wavelength dependence; photocurrent; in situ FTIR; ^15^N_2_ labeling	Local thermal effects not independently excluded	PATC^i^	M	^28^
K/Ru/TiO_2–*x*_H_*x*_	N_2_ + H_2_, 360 °C, 300 W Xe lamp (4.7 W cm^–2^)	0.1	P	Y	Charge-transfer/spillover mechanism proposed; stability changes under light	Strong plausibility of unresolved local heating at the Ru/support interface	PATC	M	^29^
K–Ru/CeO_2_	N_2_ + H_2_, 350 °C, 20 bar, 300 W Xe lamp (3.3 W cm^–2^)	19.7	Y	Y	DRIFTS, PL, photocurrent; direct vs indirect illumination	Full local-temperature matching remains experimentally difficult	PTCC	H	^30^
Ru/CeO_2_	gas, N_2_ + H_2_, 400 °C, 6.5 bar	18.0	Y	Y	Light on/off response; optical filtering; light-intensity dependence	Spatially resolved temperature diagnostics not reported	PTCC	M	^31^
Ru/C	gas, N_2_ + H_2_, 380 °C	3.5	P	Y	Equilibrium-based temperature correction; isotope studies; Raman; theory	Accuracy of inferred local catalyst temperature remains model-dependent	PTCC	M	^15^
Ru/4-TiO_2–*x*_	gas, N_2_ + H_2_, 350 °C, 300 W Xe lamp, 10 bar	0.2	Y	Y	Light-intensity dependence at fixed temperature	Local thermal gradients under irradiation not independently resolved	PTCC	L	^32^
Ru/BaTaO_2_N	N_2_ + H_2_O, 240 °C, 300 W Xe lamp (0.1 W cm^–2^)	5.9	P	—	Wavelength dependence; AQE; dark inactivity consistent with a light-driven route	Thermal contribution under elevated-temperature operation still requires careful normalization	TAPC	M	^33^
Fe_3_O_4_ films	N_2_ + H_2_, 350 °C, 300 W Xe lamp	0.4	N	N	Solar-thermal conversion behavior	Evidence supports heating rather than intrinsic kinetic modulation	PDTC	H	^34^
Fe/TiO_2_	N_2_ + H_2_, 350 °C, UVA LED (0.4 W cm^–2^)	0.1	Y	N	UVA assistance under externally heated conditions	No clear evidence of intrinsic kinetic perturbation	PATC	M	^35^
Fe-1%Al	N_2_ + H_2_, 400 °C, Xe lamp (4.7 W cm^–2^)	0.7	Y	Y	Reaction-order changes; isotope exchange; TD-DFT	Residual local thermal effects cannot be fully excluded	PTCC	H	^36^
α-Fe-110s	N_2_ + H_2_, 500 °C, 300 W Xe lamp (6.7 W cm^–2^)	0.1	Y	Y	Reaction-order changes; isotope exchange; hydrogenation of preadsorbed N*; TD-DFT	Local photothermal heterogeneity cannot be entirely excluded	PTCC	H	^37^
TiO_2–*x*_H_*y*_/Fe	N_2_ + H_2_, 495 °C, 10 bar, Xe lamp (10.2 W cm^–2^)	1.9	P	Y	EXAFS, Mössbauer, ^15^N-DRIFTS, DFT; tandem dual-zone concept	Strict local-temperature matching across hot and cool catalytic functions remains intrinsically difficult in the tandem architecture	PTCC	M	^38^
Fe-MoS_2_	N_2_ + H_2_O, 270 °C, 6 bar, solar furnace	17.0	N	—	Wavelength dependence; transient spectroscopy; ATR-FTIR; no dark NH_3_	Thermal effects assist photophysics, but matched-temperature thermal discrimination is limited	TAPC	H	^39^
AlFe/Al	N_2_ + H_2_, 360 °C, 300 W Xe lamp (4.3 W cm^–2^)	8.6	Y	Y	NAP-XPS, Raman, FTIR, DFT; strong light/dark activity disparity	High operating temperature complicates strict separation of photo- and thermo-assisted steps	TAPC^h^	H	^40^
A-CN/Fe-MXene	N_2_ + H_2_O, 80 °C, 300 W Xe lamp (0.1 W cm^–2^)	0.24	P	N	UV/vis–NIR response; photo- vs thermo- vs photothermal catalysis; ^15^N_2_ labeling; in situ DRIFTS; associative alternating pathway	Strict local thermal discrimination not established in the GVS reactor	TAPC	M	^41^
Fe-LiH	N_2_ + H_2_, 150 °C, 10 bar, 300 W Xe lamp (1.0 W cm^–2^)	0.6	P	Y	Photon-flux dependence; hydride-mediated mechanism	No strict comparison to purely thermal operation at higher accessible temperature	TAPC	L	^42^

a The table compiles key reaction-context and performance descriptors but places greater emphasis on the experimental basis used for mechanistic interpretation. For each system, the table summarizes whether matched-temperature light/dark comparisons were reported, whether illumination altered apparent kinetic signatures, what additional mechanistic evidence was provided, the main residual uncertainty, the likely dominant photothermal regime, and the confidence level associated with that interpretation. Assignments are made using the four-category framework adopted in this Review, namely photo-driven thermocatalysis, photo-assisted thermocatalysis, photothermal co-catalysis, and thermally assisted photocatalysis

b Representative reaction conditions are included only to preserve the main operating-window descriptors relevant for mechanistic interpretation; direct comparison of rates across studies remains limited by differences in temperature, pressure, irradiation conditions, hydrogen source, and reactor design.

c NH_3_ production rates are reported in mmol g^–1^ h^–1^.

d Matched-T light/dark control: **Y**, explicitly reported under nominally identical temperature; **P**, partially addressed but without rigorous local-temperature discrimination; **N**, not reported.

e Kinetic perturbation under light refers to reported changes in apparent activation energy, reaction orders, or related kinetic observables relative to dark operation.

f Additional mechanistic support includes representative examples such as optical controls, isotopic experiments, *operando* spectroscopy, excited-state probes, or theory, depending on the original report.

g Likely dominant photothermal regime: **PDTC** Photo-driven thermocatalysis; **PATC** Photo-assisted thermocatalysis; **PTCC** Photothermal co-catalysis; **TAPC** Thermally assisted photocatalysis.

h Evidence level (confidence in regime assignment): **H**, High; **M**, Moderate; **L**, Limited.

i Boundary case, approaching to PTCC regime.

### 2.2 Experimental Evidence for Mechanistic Assignment

Because multiple light-induced processes may operate simultaneously, no single experimental finding is sufficient to establish the mechanistic role of illumination in photothermal ammonia synthesis. Instead, robust assignment requires convergent evidence from complementary measurements interpreted within the thermal and kinetic constraints of the reactor.^5,10–12,16,20^

To translate this diagnostic logic into an operational framework, Table 1 summarizes the principal experimental findings and analytical tools currently used to interpret light–heat interactions in photothermal ammonia synthesis, together with their primary diagnostic value, main limitation, and the complementary evidence required for robust mechanistic assignment. Rather than serving as independent proof, these observables are most informative when evaluated in combination and under rigorously temperature-normalized conditions.

Among the most informative tests are nominally isothermal light/dark comparisons, in which the catalytic rate is evaluated under illuminated and dark conditions at the same measured catalyst temperature. In principle, a higher NH_3_ synthesis rate under illumination and equivalent thermal conditions suggests that the observed promotion cannot be explained solely by macroscopic heating. Such an interpretation, however, is only valid to the extent that the measured temperature reflects the local thermal state of the catalytic sites; otherwise, even isothermal comparisons may still conceal photothermal artifacts.^11,12^ Consequently, isothermal light/dark experiments are essential but not self-sufficient.

Apparent activation energy (*E*_a_) and reaction-order analysis can provide additional mechanistic insight, particularly when illumination induces reproducible changes in Arrhenius behavior or in the dependence of rate on N_2_, H_2_, or NH_3_ partial pressure. Such changes may suggest that illumination modifies surface coverage, the relative importance of elementary steps, or the effective rate-controlling process. Yet these signatures must be interpreted with caution. Apparent activation energies are phenomenological parameters derived from rate data over a finite temperature range and may be distorted by temperature bias, heat and mass transport effects, or shifts in adsorption equilibria unrelated to direct photoinduced chemistry.^11,12,16,18^ Likewise, altered reaction orders can confirm nonthermal contributions but do not by themselves prove carrier-mediated activation, since local thermal gradients or nonuniform surface temperatures can also redistribute coverage and influence the inferred kinetics.

Light-intensity dependence at controlled temperature is another frequent indicator. If the rate increases with photon flux while the measured catalyst temperature remains constant, this can support a nonpurely thermal contribution. However, the interpretation again depends on the reliability of the temperature measurement and on whether illumination changes the spatial heat profile within the catalyst bed without strongly affecting the average temperature readout.^11,12,18^ In addition, wavelength-dependent activity may be informative when the action spectrum tracks the optical response of the catalyst or absorber; nevertheless, wavelength dependence alone does not distinguish between photothermal conversion and direct carrier participation, because both processes may be spectrally selective.^5,7,8^

More discriminating evidence can arise from direct versus indirect illumination, optical filtering, or reactor configurations designed to decouple catalyst photoexcitation from local heat generation. Such strategies can test whether equivalent thermal profiles, when generated without direct excitation of the catalyst, reproduce the same NH_3_ synthesis rate. Likewise, chopped-light experiments may help distinguish fast reversible photoresponses from slower thermal transients, although their interpretation becomes less straightforward in systems with significant thermal inertia.^10,19,20^ Further insight can be gained from optical control experiments specifically designed to separate direct catalyst photoexcitation from predominantly thermal effects. In direct illumination experiments, the catalyst bed itself is irradiated, so that light absorption may simultaneously induce local heating and photoexcitation of the catalyst. In indirect illumination experiments, by contrast, the thermal input is generated without directly irradiating the catalyst; for instance, by using an external photothermal absorber, or optical configurations in which the catalyst is heated radiatively or conductively while its direct photoexcitation is minimized. Comparison between these two modes can test whether similar measured thermal conditions, when achieved without direct catalyst excitation, reproduce the same NH_3_ synthesis rate. If direct and indirect illumination lead to similar rates under properly matched thermal conditions, the result is more consistent with photo-driven thermocatalysis. If direct illumination produces a higher rate than indirect illumination under otherwise comparable measured temperatures, this supports a photoinduced contribution beyond heating alone, although local thermal gradients must still be carefully constrained. Optical filtering experiments can provide complementary evidence by selectively removing wavelength ranges associated primarily with catalyst photoexcitation or with photothermal heating, thereby testing whether catalytic activity tracks excitation of the catalyst rather than heat generation alone. Likewise, chopped-light experiments may help distinguish fast and reversible photoresponses from slower thermal transients. A prompt rate response that follows light on/off cycles more closely than the expected thermal relaxation may support photoinduced involvement, whereas delayed responses are more consistent with thermal effects. Even so, interpretation remains challenging in systems with significant thermal inertia or poorly resolved local temperature gradients.

Mechanistic confidence is substantially strengthened when kinetic signatures are complemented by isotopic experiments and *operando* or in situ spectroscopy. Isotope-based studies, such as ^15^N_2_ labeling or N_2_ exchange measurements, can help verify the source of nitrogen and probe whether illumination affects dissociation or subsequent hydrogenation steps. *Operando* DRIFTS, Raman, XPS, photoluminescence, transient absorption, or photocurrent measurements may reveal photoinduced charge redistribution, changes in adsorbate populations, or altered intermediate lifetimes under reaction conditions.^4,6,10^ Yet even these methods rarely provide unambiguous proof in isolation. Their main value lies in constraining which classes of mechanism remain plausible when interpreted together with temperature-normalized kinetics and appropriate controls.

For this reason, the most reliable mechanistic assignments are those supported by multiple experimental indicators: matched-temperature light/dark comparisons, careful thermal measurements, kinetic changes that persist under temperature normalization, and spectroscopic or isotopic evidence consistent with the same mechanistic interpretation. Throughout this Review, these criteria are used not as rigid binary filters, but as a practical basis for evaluating the strength of mechanistic claims across the literature.^10–12,17^

### 2.3 Sources of Conceptual Ambiguity in Mechanistic Assignment

#### 2.3.1 Temperature Measurement and Local Thermal Artifacts

Reliable temperature determination is one of the most critical and most difficult aspects of photothermal catalysis.^11,12,19^ In gas-phase photothermal reactors, the temperature read by a thermocouple, infrared camera, or pyrometer may deviate substantially from the actual temperature reached at catalytic sites. This discrepancy arises because light absorption and heat generation are often spatially nonuniform, and because the illuminated catalyst may develop axial, radial, interparticle, intraparticle, or nanoscale temperature gradients.^11,12,19^ As a result, the measured temperature may deviate from the actual temperature of the catalytic sites, and should therefore be interpreted cautiously.

For packed catalyst beds, the measured temperature may depend strongly on sensor position, bed geometry, optical penetration depth, gas flow, particle size, and thermal conductivity of the support and reactor walls.^11,12^ Even when the bulk bed temperature appears stable, illuminated particles or specific interfaces may remain hotter than their surroundings. Under these circumstances, light-induced rate enhancement can be mistakenly interpreted as nonthermal if the local temperature rise is underestimated. Conversely, Arrhenius plots constructed from temperature measurements that do not accurately reflect the actual temperature of the catalytic sites may artificially suggest lower apparent activation barriers or altered kinetics, when the dominant effect is in fact unresolved local heating.^11,12,19^

No single thermometric method fully resolves this problem. Embedded thermocouples are simple and widely used, but they probe only local points and may perturb the thermal field. Infrared thermography provides spatial information at the external surface, yet often cannot access the internal temperature of opaque catalyst beds and depends on emissivity assumptions. Optical pyrometry and blackbody-based approaches may be useful for directly illuminated surfaces, but require careful calibration and may still fail to represent the active buried interfaces in structured or packed-bed systems.^11,12,20^ More recently, *operando* structural probes such as *operando* X-ray diffraction under illumination have also been used to determine the average catalyst temperature independently of conventional contact thermometry, highlighting the value of combining complementary readouts rather than relying on a single nominal catalyst temperature.^23^

In practice, the central issue is not only how to measure temperature, but how to assess whether the measured temperature is sufficient for the mechanistic claim being made. If a study aims only to demonstrate that illumination can drive NH_3_ synthesis by heating the catalyst, approximate thermal readouts may be adequate. By contrast, if the study aims to claim intrinsic light-induced modification of the catalytic pathway, then the evidential threshold becomes much higher; local temperature bias must be constrained as far as experimentally possible, and residual uncertainty must be acknowledged explicitly.^11,12,19,21^ Accordingly, throughout this Review we treat rigorous thermal normalization not as a technical detail, but as a prerequisite for interpreting apparent photothermal synergy.

#### 2.3.2 Hot Carriers, Photogenerated Charge Carriers, and Non-Radiative Heating

A second source of conceptual ambiguity in photothermal ammonia synthesis concerns the distinction between hot carriers, photogenerated charge carriers, and nonradiative heating. These processes are often discussed together, yet they arise from different physical origins and imply different mechanistic possibilities.^4,7–9,16,24^

In plasmonic or metallic absorbers, illumination may generate nonequilibrium hot electrons and hot holes through ultrafast decay of collective electronic excitations. These carriers possess energies that deviate from the equilibrium Fermi–Dirac distribution and may, in principle, transfer to adsorbates or neighboring catalytic domains before relaxing.^16,25^ By contrast, in semiconductors and related light absorbers, photon absorption generates electron–hole pairs across the band structure, and the relevant mechanistic questions concern charge separation, migration, trapping, recombination, and interfacial transfer to adsorbed species or co-catalysts.^4,7–9^ In both cases, however, carrier relaxation ultimately leads to phonon generation and heat, so photoexcitation and photothermal heating are fundamentally linked rather than mutually exclusive.^16,20^

This distinction matters because the same irradiated catalyst may display both electronic and thermal responses on different time scales and length scales. A short-lived nonequilibrium carrier population may perturb adsorption or bond activation locally, while the subsequent thermalization may raise the local catalyst temperature and accelerate conventional thermally activated steps.^16,20,25^ As a result, the mechanistic question is rarely whether a system is purely photochemical or purely thermal, but whether photoexcitation alters the catalytically relevant pathway in a way that cannot be reduced to heating alone.

Demonstrating such a contribution remains challenging. Spectroscopic evidence of carrier generation, photocurrent, or prolonged excited-state lifetime shows that photoexcitation occurs, but does not automatically prove that those carriers participate in the rate-controlling step of NH_3_ synthesis. Conversely, observing efficient photothermal conversion does not exclude simultaneous electronic effects. Therefore, mechanistic claims involving hot-carrier or charge-carrier participation require alignment between physical evidence of excited-state generation and chemical evidence that the catalytic pathway is modified in a manner consistent with those excited states.^16,24^

For this reason, the present Review uses the terms photo-assisted thermocatalysis and thermally assisted photocatalysis in an operational sense. They are not intended to imply complete separation of heat and photoexcitation, but rather to identify which contribution appears to dominate the mechanistically relevant step under the reported conditions. This distinction is especially important when comparing Ru- and Fe-based systems, where illumination may interact with different rate-controlling constraints and therefore become mechanistically visible in different ways.^6^

To apply the diagnostic framework introduced above, Table 2 reviews representative photothermal ammonia synthesis systems in terms of the specific evidence used to support mechanistic interpretation. Rather than listing catalyst performance alone, the table links each system to the corresponding temperature-normalized controls, reported kinetic perturbations under illumination, additional mechanistic support, and the main residual uncertainty that limits mechanistic confidence. To preserve continuity with the comparative discussion developed in the following sections, systems are grouped by the dominant metal platform (Ru or Fe), while their order within each group follows the likely dominant photothermal regime and, secondarily, the strength of the available mechanistic evidence. Within this organization, the regime sequence considered is photo-driven thermocatalysis (PDTC), photo-assisted thermocatalysis (PATC), photothermal co-catalysis (PTCC), and thermally assisted photocatalysis (TAPC), although not every metal platform is represented in all four categories. Such studies are also discussed in more detail in the following sections.

## 3 Mechanistic Interpretation Of Ru-Based Photothermal Ammonia Synthesis

Ruthenium-based catalysts remain the most extensively studied and, in many cases, the most mechanistically informative systems in photothermal ammonia synthesis. This prominence reflects not only the high intrinsic activity of Ru for thermocatalytic NH_3_ synthesis, but also the fact that Ru-based platforms have provided some of the clearest opportunities to examine whether illumination merely accelerates an already thermally activated process or instead perturbs kinetically relevant steps in ways that cannot be reproduced by external heating alone. At the same time, Ru literature also illustrates the central challenge emphasized throughout this Review: similar catalyst families may display comparable signatures under illumination, yet differ substantially in the strength of the evidence supporting their assignment. Thus, the discussion below does not treat Ru-based systems as belonging to rigid regime classes. Instead, each case is examined in terms of three linked questions: (i) what type of experimental discrimination between thermal and photoinduced contributions was actually performed, (ii) whether illumination altered the effective kinetic response beyond what can be explained by measured bulk heating, and (iii) what residual uncertainty remains, especially regarding unresolved local thermal effects. This approach is particularly important for Ru systems supported on defective oxides, where charge-transfer effects, photothermal heating, and interfacial thermal gradients may coexist under reaction conditions.

### 3.1 Photo-Driven Thermocatalysis in Ru-Based Systems

Ru-based systems within the photo-driven thermocatalysis category exemplify cases where illumination clearly improves catalytic performance, yet the available evidence indicates that this improvement can be rationalized primarily through heat generation and heat management rather than through a demonstrable change in the intrinsic reaction mechanism.

A representative example of this category is the Cs-promoted Ru/MgO system reported by Li and co-workers,^26^ in which illumination was used to generate strong axial thermal gradients across the catalyst bed. Under concentrated blue LED irradiation, the authors observed markedly enhanced NH_3_ synthesis and interpreted this effect using an “equivalent temperature” concept, explicitly recognizing that the catalyst bed was not uniformly heated (Figure 1a). No matched-temperature light/dark comparison at rigorously equivalent local temperatures was reported, and no convincing evidence was provided that illumination altered the intrinsic kinetic response of the catalyst. Instead, the enhanced performance under illumination was convincingly attributed to thermal-gradient engineering, whereby the hotter upstream region promotes N_2_ dissociation, whereas cooler downstream regions help suppress NH_3_ decomposition. In the framework adopted here, this system is therefore best interpreted as a high-confidence case of photo-driven thermocatalysis, where light acts primarily as a spatially resolved heat source rather than as a driver of nonthermal surface chemistry.

**Figure 1:**
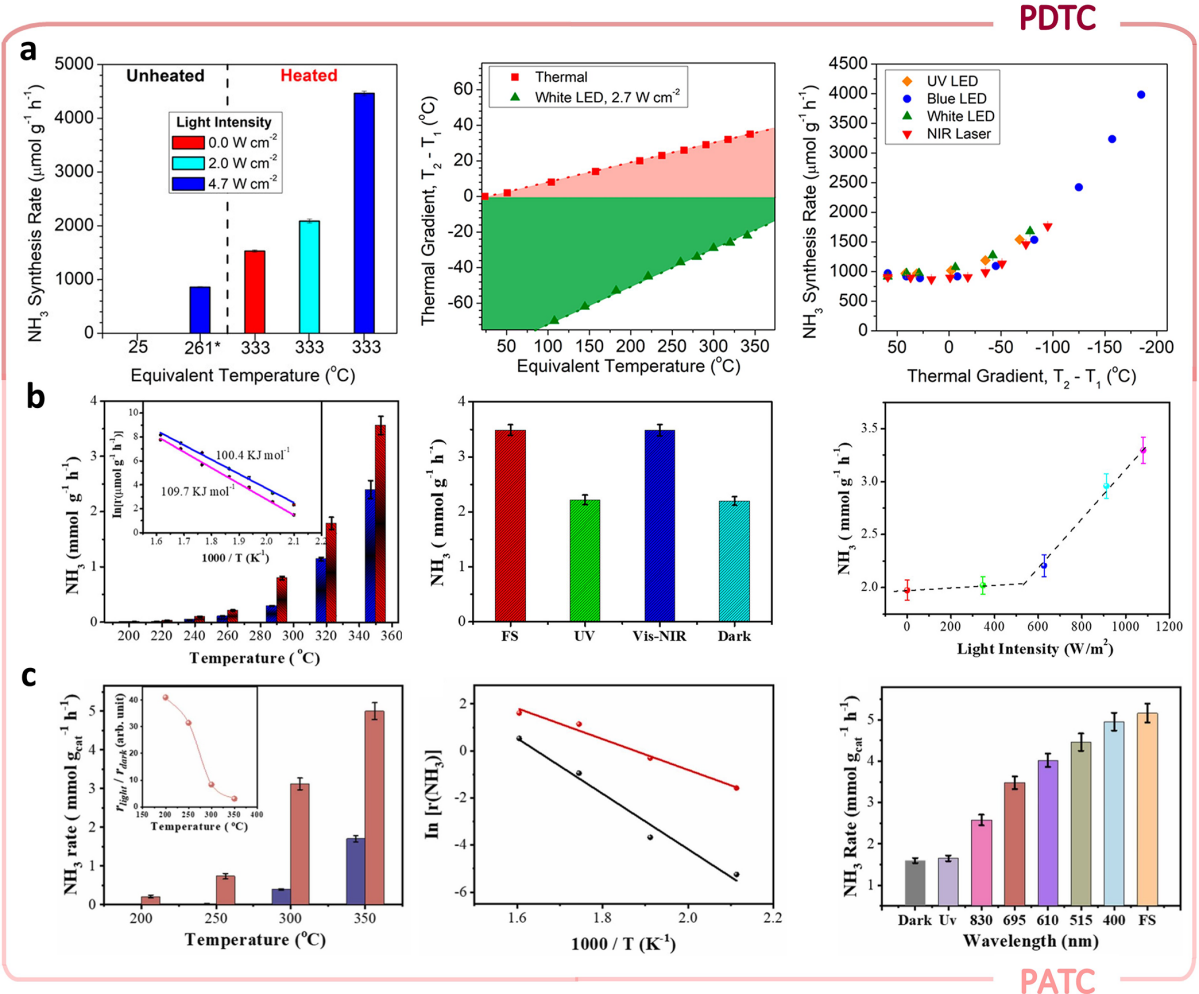
Representative Cs-promoted Ru-based systems in the PDTC and PATC regimes. (a) Cs–Ru/MgO, illustrating illumination-induced axial thermal gradients and the equivalent-temperature concept used to rationalize enhanced NH_3_ formation through thermal-gradient engineering rather than intrinsic pathway modification. Adapted from ref 26 with permission (Copyright 2019 American Chemical Society). (b) Cs–Ru@SrTiO_3_, showing higher activity under visible/NIR irradiation together with similar apparent activation energies in the light and dark. Adapted from ref 27 with permission (Copyright 2022 American Chemical Society). (c) Cs–Ru/ZrO_2_, showing illumination-enhanced NH_3_ synthesis accompanied by lower apparent activation energy. Adapted from ref 28 with permission (Copyright 2023 Elsevier).

### 3.2 Photo-Assisted Thermocatalysis in Ru-Based Systems

The earliest case within this category corresponds to the K/Ru/TiO_2–*x*_H_*x*_ study of Mao and co-workers.^29^ This catalyst displayed illumination-enhanced NH_3_ synthesis together with a proposed mechanism involving charge transfer and hydrogen spillover. The authors reported changes in apparent activation energy and reaction orders under illumination, suggestive of intrinsic light–heat coupling effects. However, because the system operated under concentrated irradiation and without rigorous discrimination of interfacial thermal gradients, the possibility that localized heating contributed substantially to the inferred kinetic perturbation remains particularly strong. Such effects introduce inherent uncertainty in distinguishing intrinsic kinetic modification from spatially confined photothermal heating. For this reason, K/Ru/TiO_2–*x*_H_*x*_ is best treated as a moderate-confidence case of photo-assisted thermocatalysis.

Later on, Peng and co-workers^27^ reported a Cs-promoted Ru/SrTiO_3_ system active under visible and near-infrared (NIR) irradiation (Figure 1b). In this study, matched-temperature light/dark comparisons showed higher activity under illumination. However, the apparent activation energies under light and dark conditions remained essentially similar, indicating that the same rate-determining step governs light-assisted and thermal reactions in the reaction mechanism. Wavelength-dependent studies showed a more pronounced enhancement under NIR irradiation, consistent with light absorption by Ru rather than by the SrTiO_3_ support. Although a photo-induced hot carrier contribution was proposed, the kinetic similarity between illuminated and dark conditions suggests that illumination does not measurably alter the intrinsic reaction pathway. Instead, the data are most consistent with localized photothermal acceleration at Ru active sites, with SrTiO_3_ primarily facilitating heat localization through its basicity and low thermal conductivity. Under the present framework, this system is therefore more conservatively interpreted as photo-assisted thermocatalysis with moderate confidence: illumination likely enhances catalysis through localized photothermal acceleration at Ru active sites, possibly accompanied by a secondary hot-carrier contribution, rather than as a clear intrinsic modification of the catalytic pathway.

One year later, Peng and co-workers^28^ reported a more detailed kinetic analysis for Cs-promoted Ru/ZrO_2_ (Figure 1c). In contrast to the Cs–Ru/SrTiO_3_ system, Arrhenius analysis revealed a markedly lower apparent activation energy under illumination accompanied by measurable changes in reaction orders for both N_2_ and H_2_, indicating altered surface kinetics. Notably, the Arrhenius plots remained linear over the same temperature window in both dark and illuminated conditions, consistent with an overall thermally activated reaction. On the other hand, wavelength-dependent activity, photocurrent measurements, isotopic ^15^N_2_ labeling, and in situ FTIR spectroscopy were collectively interpreted as evidence for hot-electron-assisted N_2_ activation. These features make Ru–Cs/ZrO_2_ more than a purely photo-assisted thermocatalytic case, because illumination does not only enhance the rate but also modifies kinetics and provides optical and spectroscopic signatures consistent with photoinduced participation. However, the system is not assigned here to definitive photothermal co-catalysis because independent constraints on local catalyst temperature, direct-versus-indirect illumination discrimination, or equivalent thermal-profile controls were not reported, leaving localized photothermal effects as a plausible contributor to the apparent kinetic changes. It is therefore best described as a PATC/PTCC boundary case.

### 3.3 Photothermal Co-Catalysis in Ru-Based Systems

Building upon the defect-engineered Ru/TiO_2–*x*_ platform, Chang and co-workers^32^ reported a Ru/4-TiO_2–*x*_ catalyst exhibiting higher NH_3_ synthesis rates under illumination at identical macroscopic temperatures. The enhancement was particularly pronounced below 300 °C, where kinetic limitations are more severe, suggesting that illumination modifies the effective reaction pathway under kinetically constrained conditions. In addition to temperature-controlled comparisons, a clear dependence of ammonia synthesis rate on light intensity was observed at fixed bulk temperature providing further evidence that the rate enhancement cannot be attributed solely to macroscopic heating effects. Apparent activation energies were reduced under illumination, and reaction orders with respect to both N_2_ and NH_3_ were altered relative to dark operation. The reported kinetic features indicate that illumination affects intrinsic reaction energetics, likely through photoinduced charge redistribution at the Ru/TiO_2–*x*_ interface, promoting different reaction pathways from the purely thermal route. From a mechanistic standpoint, experimental findings are consistent with photothermal co-catalysis, even though they are not supported by spectroscopic evidence. Besides, the unresolved local thermal gradients at the illuminated Ru/TiO_2–*x*_ interface remain a plausible contributor, and therefore the most rigorous assignment is low confidence photothermal co-catalysis.

Feng and co-workers^31^ reported isolated Ru sites on CeO_2_ that achieved substantially higher NH_3_ synthesis rates under illumination than under dark thermal operation at fixed reaction temperature (Figure 2a). Reversible light on/off cycling produced reproducible changes in activity under externally controlled thermal conditions. Although detailed spatial temperature diagnostics were not reported, the persistence of enhanced rates under nominally constant bulk temperature suggests that the observed promotion cannot be attributed solely to macroscopic photothermal heating. Light-intensity-dependent measurements further showed a positive correlation between photon flux and catalytic performance. Importantly, when a liquid optical filter was employed to suppress photothermal contributions, the activity enhancement remained detectable, supporting a role for photoexcitation beyond simple heat localization. A reduced apparent activation energy under illumination was interpreted by the authors as evidence that light optimizes the reaction pathway, with implications for the rate-determining step. While the overall process remains thermally activated, the combination of isothermal light/dark controls, light-intensity dependence, activation energy reduction, and optical filtering experiments provides compelling evidence, within the limits of the reported thermal diagnostics, that illumination intrinsically modifies surface reaction energetics. Thus, this study supports assignment to photothermal co-catalysis with high confidence, although full spatial resolution of local catalyst temperature was not reported.

**Figure 2:**
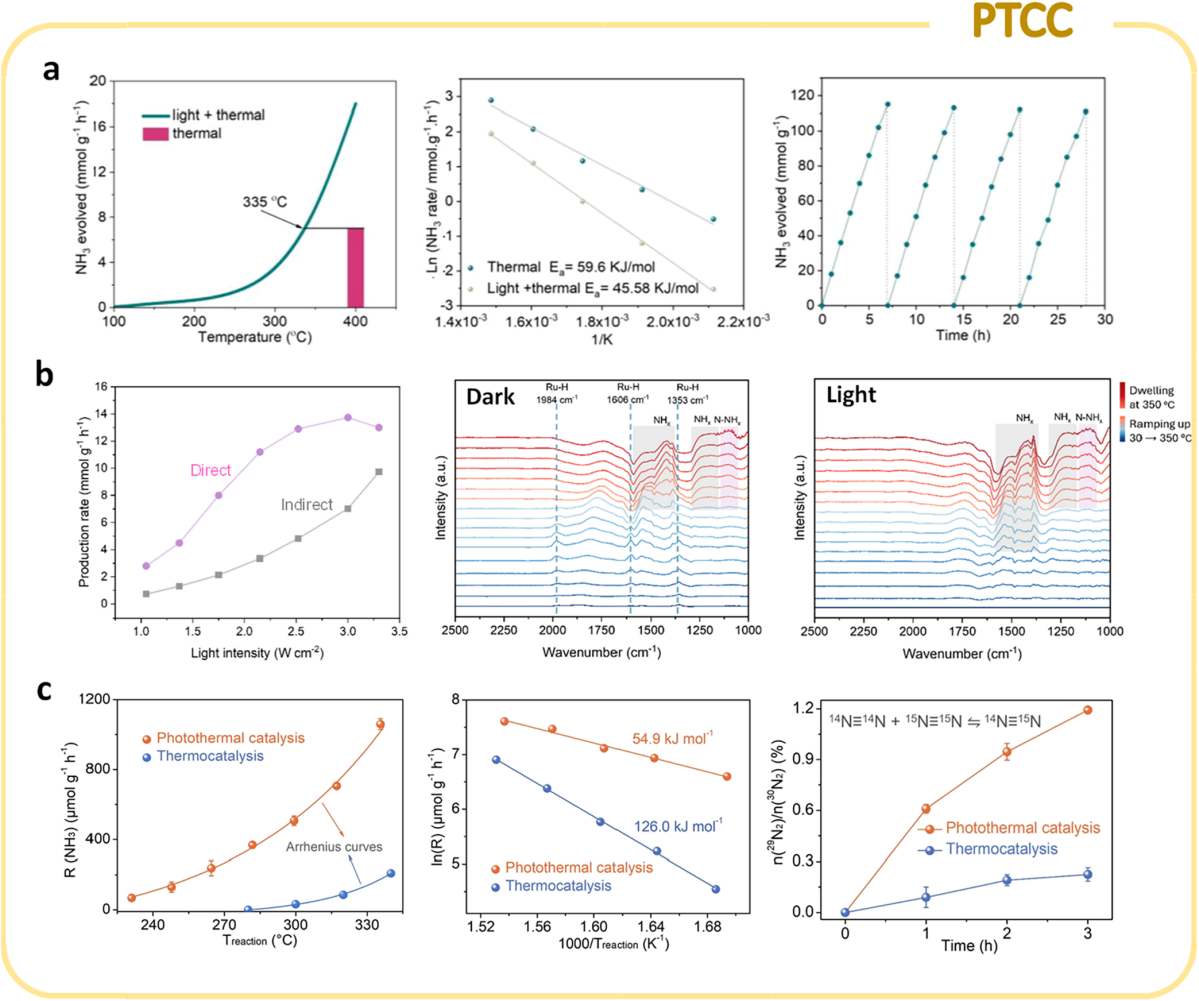
Representative Ru-based systems assigned to photothermal co-catalysis. (a) Ru/CeO_2_, illustrating enhanced NH_3_ formation under illumination, reversible light on/off response, and reduced apparent activation energy relative to dark thermal operation. Adapted from ref 31 with permission (Copyright 2024 Wiley-VCH GmbH). (b) K–Ru/CeO_2_, showing direct-versus-indirect illumination discrimination together with in situ spectroscopic evidence for illumination-induced electronic redistribution at the Ru/CeO_2_ interface. Adapted from ref 30 with permission (Copyright 2025 Scilight). (c) Ru/C, illustrating equilibrium-based temperature correction, lower apparent activation energy under photothermal operation, and isotope-supported evidence for altered N_2_ dissociation and hydrogenation kinetics. Adapted from ref 15 with permission (Copyright 2023 Wiley-VCH).

A closely related but even more methodologically explicit case was recently reported by Sousa and co-workers^30^ for K-promoted Ru/CeO_2_. In this work, systematic light/dark comparisons were conducted at identical reaction temperatures and pressures, revealing consistently higher NH_3_ production rates under illumination. Kinetic analysis further showed a reduced apparent activation energy and modified reaction orders under illumination, consistent with a light-induced shift in surface reaction energetics that cannot be reproduced by external heating alone. Particularly important was the use of a direct versus indirect illumination strategy (Figure 2b), in which a separate photothermal absorber reproduced the thermal input while suppressing direct photoexcitation of the catalyst. Under indirect illumination, NH_3_ formation exhibited an exponential dependence on light intensity, characteristic of purely thermally driven kinetics, while direct illumination led to a linear rate dependence consistent with a photon-assisted contribution. The divergence between direct and indirect illumination at comparable macroscopic temperature provided one of the clearest available indications that photoinduced effects contribute beyond simple heat generation, modulating reaction kinetics even within a high-temperature regime. This methodology provides one of the clearest experimental attempts to isolate electronic effects from photothermal heating in Ru-based systems. Within the classification framework adopted here, this system aligns most closely with a high-confidence photothermal co-catalysis regime, as illumination induces measurable kinetic modifications beyond those attributable to photothermal heating alone.

A conceptually distinct but still important case is the Ru/C catalyst studied by Bian and co-workers.^15^ Here, the authors attempted to estimate the actual catalyst temperature under illumination from equilibrium conversion and used this corrected temperature to separate the contribution of local heating from that of hot-electron effects. On this basis, the photothermal route showed a markedly lower apparent activation energy than the dark thermal route (Figure 2c), while isotope experiments, in situ Raman measurements, and theoretical analysis were used to support accelerated N_2_ dissociation and altered hydrogenation kinetics under illumination. Although the inferred catalyst temperature remains model-dependent and therefore cannot be regarded as a direct local thermometric measurement, this work nevertheless provides unusually strong evidence that illumination perturbs the effective kinetics beyond what can be rationalized by nominal heating alone. Within the present framework, Ru/C is therefore also best interpreted as photothermal cocoupling with high confidence, although on the basis of indirect temperature correction rather than direct local thermal probing.

### 3.4 Thermally Assisted Photocatalysis in Ru-Based Systems

A different mechanistic picture emerges for the Ru-supported BaTaO_2_N oxynitride system reported by Li and co-workers,^33^ which is best interpreted as a case of thermally assisted photocatalysis. In this study, ammonia was produced from N_2_ and water under simulated solar irradiation at elevated temperature, with no detectable NH_3_ formation in the absence of light and with a strong dependence of activity on excitation wavelength and quantum efficiency across the visible region. Time-resolved photoluminescence showed prolonged charge-carrier lifetime, and the authors proposed that photogenerated electrons are preferentially trapped on Ru nanoparticles while holes remain on the oxynitride support, thereby promoting charge separation and facilitating N_2_ reduction. At the same time, the system was deliberately operated at elevated temperature, and heating was proposed to promote oxygen-vacancy formation, enhance oxygen evolution kinetics, and improve the overall catalytic performance. The available evidence is therefore more consistent with a photochemical-dominant pathway in which photoexcitation governs the key activation chemistry, while heat plays a secondary but still important role in facilitating carrier utilization and subsequent surface redox steps. Even so, because the study does not provide strict matched-temperature light/dark discrimination, the most rigorous assignment is thermally assisted photocatalysis with moderate confidence.

Collectively, the Ru-based literature therefore spans much of the diagnostic range proposed in this Review. Some systems are best explained by illumination-induced heat management, others provide strong evidence that light perturbs an otherwise thermally activated Ru pathway, others clear support that thermochemical and photoinduced pathways contribute simultaneously and cooperatively, and still others, such as Ru/BaTaO_2_N, point toward thermally assisted photocatalysis, in which photoexcitation governs the key activation chemistry while elevated temperature assists surface redox turnover and overall efficiency.

## 4 Mechanistic Interpretation of Fe-Based Photothermal Ammonia Synthesis

Compared with Ru-based systems, Fe-based photothermal ammonia synthesis exhibits a broader mechanistic spread and, in many cases, a higher degree of interpretive complexity. This is partly because Fe catalysts operate under a different baseline kinetic landscape, in which N_2_ dissociation barriers, ammonia inhibition, and active-site structure play a more prominent role in determining overall reactivity. In the present framework, Fe-based systems are therefore discussed using the same diagnostic criteria applied to Ru-based catalysts: the key questions are whether illumination perturbs the effective kinetic response beyond nominal heating, what type of mechanistic evidence supports that interpretation, and what residual uncertainty remains regarding unresolved local thermal effects.

### 4.1 Photo-Driven Thermocatalysis in Fe-Based Systems

The Fe-based systems assigned here to photo-driven thermocatalysis are those for which illumination clearly enhances catalytic performance, but the available evidence does not support a measurable change in the intrinsic reaction pathway beyond heat generation or photothermal acceleration. In these cases, the mechanistic interpretation rests less on illumination-induced perturbation of kinetic observables and more on the absence of compelling evidence for such perturbation once the thermal role of light is considered.

A representative example is the Fe_3_O_4_ nanostructure film system reported by Fu and co-workers.^34^ In this study, Fe_3_O_4_ films were proposed as efficient solar-thermal conversion materials for ammonia synthesis, and the reported enhancement was interpreted primarily in terms of the material’s ability to absorb light and convert it into heat. The work is therefore important as a demonstration that iron-based materials can serve as effective solar-thermal absorbers for NH_3_ synthesis, but it does not provide matched-temperature light/dark discrimination or kinetic evidence indicating that illumination alters the intrinsic catalytic pathway. Within the present framework, this system is most appropriately classified as photo-driven thermocatalysis with high confidence.

### 4.2 Photo-Assisted Thermocatalysis in Fe-Based Systems

The TiO_2_-based Fe system discussed by Valenzuela and co-workers^35^ represents a plausible photo-assisted thermocatalysis case. In this work, Fe-TiO_2_ was evaluated under UV-A irradiation at ambient pressure, and dual photonic/thermal excitation increased the NH_3_ production rate while also enabling operation at lower temperature than under purely thermal conditions. The study reported that, unlike Ru/TiO_2_, Fe-TiO_2_ showed no significant illumination-induced change in apparent activation energy, and the authors accordingly suggested that a light-induced localized heat-delivery mechanism predominates in this system. Taken together, these results support a catalyst that benefits from light under thermally relevant conditions but without clear evidence that illumination intrinsically modifies the reaction pathway, making PATC the most consistent interpretation, although with moderate mechanistic confidence.

### 4.3 Photothermal Co-Catalysis in Fe-Based Systems

Among the representative Fe-based studies considered here, the most rigorous demonstration of light–heat coupling on iron is provided by the tailored α-Fe metallic material reported by Yang and co-workers.^37^ This catalyst intrinsically lowered the thermal barrier for N_2_ activation in the absence of promoters or supports. Here, strictly isothermal light/dark comparisons (Figure 3a) were accompanied by changes in reaction orders, isotope-exchange experiments, and hydrogenation studies of preadsorbed nitrogen species, together with theoretical analysis supporting selective hot-electron transfer from Fe to adsorbed N_2_ and modified adsorption energetics of NH_*x*_ intermediates. Particularly important is that the study does not rely on activity enhancement alone, but shows that illumination intrinsically modifies elementary reaction energetics while ammonia synthesis remains fundamentally thermally activated.

**Figure 3:**
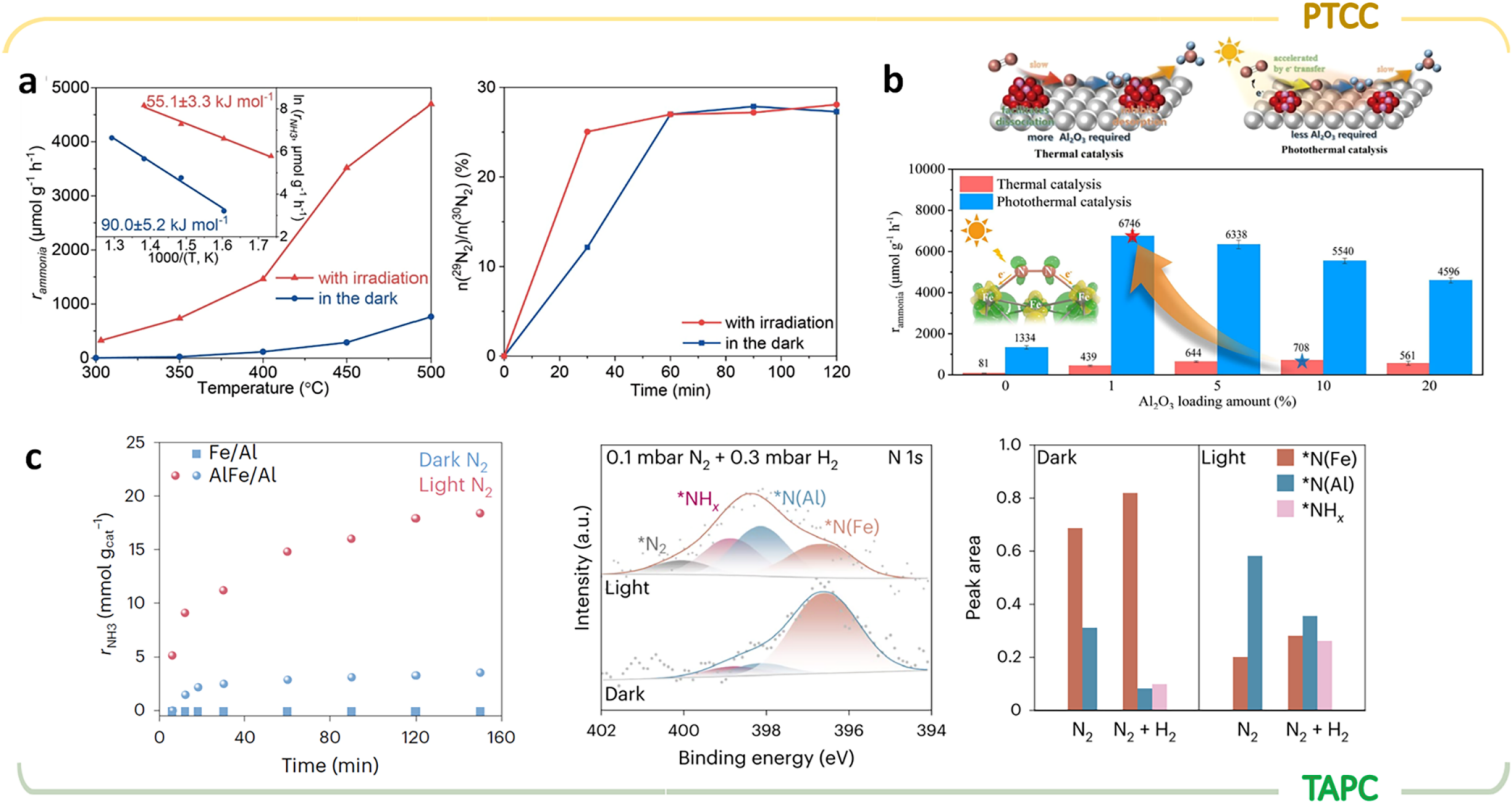
Representative Fe-based systems spanning photothermal co-catalysis and thermally assisted photocatalysis. (a) α-Fe-110s, showing strictly isothermal light/dark kinetic discrimination and reduced apparent activation energy under illumination, consistent with illumination-induced modification of elementary steps on stepped Fe surfaces. Adapted from ref 37 with permission (Copyright 2024 Wiley-VCH). (b) Fe-1%Al, illustrating the dependence of thermal and photothermal NH_3_ synthesis rates on Al_2_O_3_ loading, together with the different optimum promoter requirement under thermal and illuminated conditions. Adapted from ref 36 with permission (Copyright 2025 Elsevier). (c) AlFe/Al, illustrating strong light/dark activity disparity and spectroscopic evidence for photoinduced nitrogen spillover between Fe and Al sites. Adapted from ref 40 with permission (Copyright 2026 Springer Nature).

Another representative example is the alumina-modified iron system reported by Ding and co-workers.^36^ In this study, illumination enhanced NH_3_ synthesis under thermally relevant conditions (Figure 3b) and was accompanied by measurable changes in the apparent activation energy and reaction orders, indicating that illumination alters the effective rate-determining regime rather than merely accelerating thermal rates. Isotopic ^28^N_2_/^30^N_2_ exchange experiments and theoretical studies evidenced enhanced N≡N activation and subsequent hydrogenation steps, consistently supporting illumination-driven modification of elementary steps under nominally isothermal conditions. The importance of this work lies in showing that, for Fe-based catalysts, illumination may alter the effective reaction pathway not by bypassing thermal activation, but by reshaping the balance between adsorption, dissociation, and hydrogenation steps on an already active surface.

A conceptually distinct Fe-based strategy was reported by Mao and co-workers^38^ through a dual-temperature-zone ammonia synthesis using a plasmonic TiO_2–*x*_H_*y*_/Fe catalyst. In this work, solar irradiation was used to sustain a tandem configuration in which spatially differentiated catalyst functions operating at different effective temperatures jointly contributed to NH_3_ synthesis beyond the conventional thermal equilibrium limit. The hot Fe component was associated with N_2_ activation and dissociation, whereas the cooler TiO_2–*x*_H_*y*_ component contributed to subsequent hydrogenation chemistry. EXAFS, Mössbauer analysis, ^15^N-DRIFTS, and DFT were all consistent with a coupled thermo-photochemical pathway based on the cooperative interplay between these distinct yet connected catalytic functions. At the same time, the architecture makes strict local-temperature matching intrinsically difficult, since the mechanistic concept itself relies on spatially differentiated thermal functions rather than on a single uniformly heated catalytic state. Accordingly, this system is best regarded as a representative Fe-based example of photothermal co-catalysis with moderate confidence: the available evidence strongly supports light–heat interplay beyond uniform photothermal heating, but the absence of reaction-order analysis and strictly isothermal local-temperature discrimination prevents a more definitive mechanistic assignment.

### 4.4 Thermally Assisted Photocatalysis in Fe-Based Systems

A further subset of Fe-based systems is more consistently interpreted as thermally assisted photocatalysis, namely those in which illumination appears to govern the key activation chemistry while elevated temperature primarily improves carrier utilization, surface transport, or downstream reaction kinetics.

One of the clearest examples is the Fe on MoS_2_ system reported by Zheng and co-workers.^39^ In this study, ammonia formation was observed in aqueous conditions under concentrating visible-light irradiation. The absence of detectable NH_3_ formation in the dark, even at elevated temperature, and a strong wavelength-dependence activity strongly supports photon-governed activity, rather than bulk heating. Transient spectroscopy, in situ ATR-FTIR, and theoretical calculations supported an associative hydrogenation pathway in which photoexcited states are directly involved in nitrogen activation, while thermal input mainly facilitates photoinduced electron transfer to adsorbed nitrogen. Because the available evidence points consistently toward a light-driven route, this system is best classified as thermally assisted photocatalysis with high confidence, in which heat modulates photophysical processes rather than directly redefining surface reaction kinetics.

A similarly strong but mechanistically distinct case is the AlFe/Al dual-site catalyst reported by Li and co-workers.^40^ In this system, the large activity disparity between illuminated and dark conditions (Figure 3c), together with thermocatalytic rates that are two to 3 orders of magnitude lower at the same temperature, indicates that photoexcitation plays a dominant role in enabling ammonia synthesis. Kinetic analysis further revealed a marked decrease in apparent activation energy under illumination, accompanied by changes in reaction orders consistent with enhanced N_2_ activation and reduced NH_3_ inhibition relative to thermocatalytic operation. *Operando* NAP-XPS, Raman, FTIR, and DFT analysis supported a photoinduced nitrogen spillover mechanism in which Fe sites activate N_2_, while adjacent Al sites act as secondary adsorption centers for dissociated N species, facilitating subsequent hydrogenation and NH_3_ desorption. These results suggest that photocarriers participate directly in the key activation chemistry, whereas photothermal heating mainly assists the overall reaction kinetics under elevated-temperature conditions. Accordingly, AlFe/Al is best interpreted as thermally assisted photocatalysis with high confidence, although its strong coupling between photo- and thermo-assisted contributions places it close to the boundary with photothermal co-catalysis.

A more moderate-confidence example is the A-CN/Fe-MXene aerogel system reported by Tang and co-workers.^41^ In this work, NH_3_ synthesis from N_2_ and H_2_O was studied in a gas-vapor–solid photothermal reactor designed to reduce water-induced light attenuation and improve reactant transport. Enhanced photocurrent under combined light and heat, ^15^N_2_ labeling, and in situ DRIFTS were consistent with an associative alternating pathway under irradiation. Comparisons between photocatalytic, thermocatalytic, and photothermal operation further indicated that light plays the dominant role, whereas heat assists by facilitating carrier utilization and surface reaction kinetics. Negligible NH_3_ formation under purely thermal conditions likewise supports a fundamentally light-driven pathway rather than one sustained primarily by heating alone. Even so, because the system was not subjected to strict matched-temperature light/dark discrimination with rigorous local thermometry, the partition between photoinduced and thermal contributions remains only partially resolved. Accordingly, A-CN/Fe-MXene is best interpreted as thermally assisted photocatalysis with moderate confidence.

A distinct hydride-mediated photothermal regime was recently reported by Zhang and co-workers^42^ using a Fe-LiH composite. In this study, illumination triggered NH_3_ formation at relatively low bulk temperature, whereas it was undetectable under dark operation at the same apparent temperatures, indicating that light plays a critical role in N_2_ activation under these conditions. The NH_3_ synthesis rate also showed an approximately linear dependence on photon flux at fixed temperature, together with reduced apparent activation energies under illumination and selective changes in reaction orders consistent with weakened hydrogen constraint and attenuated NH_3_ inhibition. Spectroscopic measurements further supported a hydride-mediated mechanism in which light promotes reductive elimination of hydridic hydrogen from LiH, generating electron-rich Fe-LiH_1–*x*_ sites that enable LiNH_2_ formation and subsequent NH_3_ release. Even so, because the thermal reference experiments were limited to the same low-temperature window and strict temperature-matched light/dark comparisons were not established, the relative roles of intrinsic electronic effects and localized photothermal heating remain insufficiently resolved. Accordingly, Fe-LiH is best regarded as a limited-confidence case of thermally assisted photocatalysis.

Overall, the Fe-based literature confirms that photothermal ammonia synthesis cannot be interpreted through a single mechanistic template. Compared with Ru-based systems, Fe catalysts often display greater sensitivity to active-site structure, nitrogen activation barriers, and catalyst architecture, which makes mechanistic interpretation both richer and more challenging. In this sense, Fe-based photothermal catalysts do not simply represent an alternative metal platform, but a particularly informative family for understanding how illumination becomes mechanistically visible when it interacts with a different kinetic landscape. The comparison between Ru- and Fe-based systems therefore suggests that the mechanistic role of light is not determined by metal identity alone, but by the interplay between intrinsic catalytic constraints, catalyst architecture, interfacial charge dynamics, and the extent to which illumination perturbs the kinetically relevant elementary step. This broader perspective is developed in the following section.

## 5 Structure-, Interface-, and Metal-Dependent Kinetic Constraints

The analysis above shows that the mechanistic role of illumination in photothermal NH_3_ synthesis is not dictated by metal identity alone. Rather, it depends on whether light absorption, heat generation, charge redistribution, and the kinetically relevant elementary step occur at the same catalytic interface.

For Ru-based catalysts, the most useful trends are materials-related rather than purely categorical. Ru provides a highly active thermocatalytic phase for N_2_ dissociation and H_2_ activation, so the role of illumination is strongly conditioned by how the support and promoter modify Ru dispersion, electron density, hydrogen coverage, and heat localization. Alkali-promoted Ru catalysts supported on basic or thermally insulating oxides can benefit from enhanced local heat delivery and favorable catalyst-bed thermal gradients, especially when the support mainly improves Ru dispersion, basicity, or heat confinement without providing clear redox participation in the catalytic reaction. In such materials, light-induced enhancement can remain largely associated with photothermal heating or thermal-profile control, even if the support is optically or electronically responsive. By contrast, Ru supported on reducible or defect-rich oxides introduces additional interfacial degrees of freedom. Oxygen vacancies, Lewis acid–base sites, metal–support charge transfer, and promoter-induced electron donation can all modify N_2_ adsorption, hydrogen coverage, and NH_*x*_ hydrogenation at the Ru/support interface. This is particularly relevant for CeO_2_- and TiO_2–*x*_-based materials, where redox-active supports and defective interfaces may couple light absorption, local heat generation, and electronic redistribution near the same catalytic boundary. However, this structural motif does not by itself define a synergistic regime, because the same interfacial localization that favors electronic perturbation also favors unresolved nanoscale heating. Therefore, among Ru catalysts, the progression from heat-dominated behavior to stronger light–heat coupling is better understood as a consequence of increasing functional integration between Ru sites, promoters, support defects, and optical absorption, rather than as a simple consequence of using a reducible oxide support. A distinct situation is found when Ru is deposited on a semiconductor or oxynitride that acts primarily as the photoabsorber and charge-separating phase; in that case, Ru behaves less as the thermocatalytic center of a conventional Ru catalyst and more as an electron-accepting or reduction co-catalyst, shifting the material design toward a photocatalysis-dominant architecture.

Fe-based catalysts show a different set of kinetic and structural constraints. Iron oxides or Fe-containing materials with strong broadband absorption can act primarily as solar-thermal absorbers, where morphology, optical absorption, and heat generation dominate the catalytic response. In such cases, nanostructuring or film architecture mainly improves light harvesting and thermal management, while the catalytic pathway remains close to conventional thermocatalysis unless additional interfacial or kinetic evidence indicates otherwise. Metallic Fe catalysts with controlled surface structure provide a different situation, because steps, low-coordination Fe atoms, and crystallographic anisotropy can directly alter N_2_ adsorption and dissociation. When illumination interacts with these structurally sensitive sites, the effect of light may become visible through changes in N_2_ activation, NH_*x*_ binding, hydrogenation barriers, or NH_3_ desorption. Electronic modification by Lewis-acid sites, such as alumina-derived sites, can further tune Fe electron density, stabilize nitrogen-containing intermediates, and change the balance between N_2_ dissociation, hydrogenation, and ammonia desorption. These materials therefore illustrate how relatively small changes in Fe local coordination or promoter environment can make light-induced perturbations more detectable than in less structurally defined Fe catalysts.

Fe-based heterostructures introduce additional interfacial functions that are less common in conventional Fe thermocatalysis. Sulfide motifs, MXene-derived interfaces, Fe–Al dual sites, and hydride-containing Fe-LiH systems can provide secondary adsorption sites, charge-transfer channels, nitrogen spillover pathways, or hydride-mediated hydrogen transfer. In these architectures, Fe is not only a site for N_2_ activation; it operates as part of a multifunctional interface in which light absorption, carrier generation, hydrogen transfer, and nitrogen intermediate stabilization may occur on different but coupled components. This can shift the material behavior away from simple heat-assisted Fe thermocatalysis toward photocatalysis-dominant or strongly coupled light–heat regimes, depending on whether photoexcitation primarily modifies an already thermal Fe pathway or directly enables the key nitrogen activation chemistry. Thus, for Fe-based materials, the decisive design variable is the extent to which active-site geometry, electronic promotion, and secondary interfacial functions cooperate to overcome the N_2_ activation barrier and the sensitivity of Fe surfaces to hydrogen and ammonia coverage.

## 6 Recommended Reporting Metrics and Minimum Evidence Standards

The diversity of mechanistic interpretations discussed above makes clear that progress in photothermal ammonia synthesis depends not only on new catalyst designs, but also on more consistent reporting practices. At present, comparison across studies remains difficult because catalytic performance is often reported under substantially different reaction conditions, reactor geometries, analytical methods, and temperature-measurement protocols.^6,10–12^ As a result, high NH_3_ production alone does not necessarily provide mechanistic clarity, and similar catalytic trends may be interpreted very differently depending on the extent of thermal normalization and supporting evidence. For this reason, the field would benefit from a more explicit distinction between performance reporting and mechanistic reporting, together with a minimum evidence standard for assigning the role of illumination (Figure 4).

**Figure 4:**
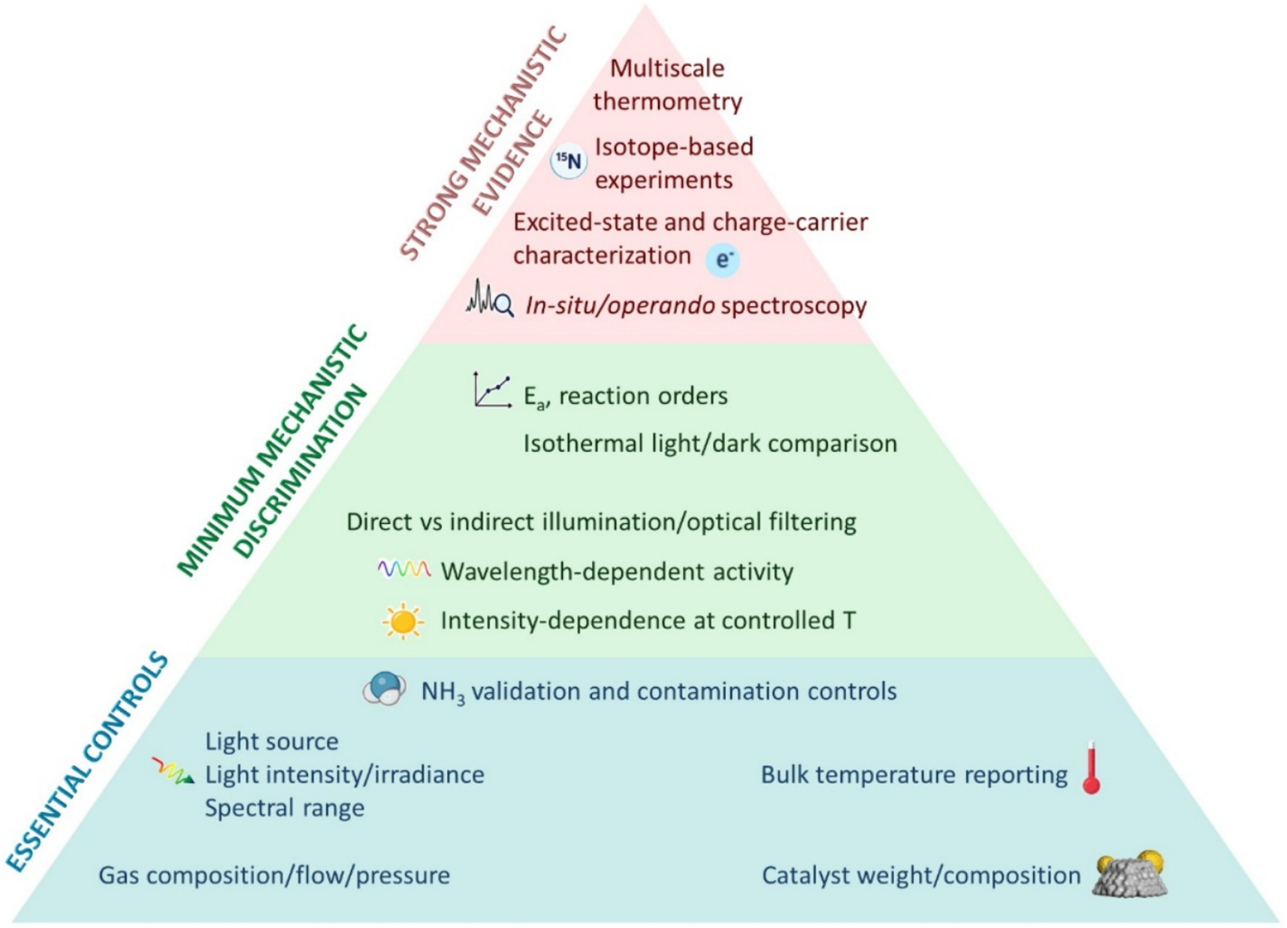
Increasing experimental evidence for mechanistic interpretation in photothermal ammonia synthesis.

At the level of catalytic performance, the minimum information reported should include catalyst composition (including co-catalyst or dopant loading, support, promoter content, etc.), catalyst mass, nitrogen and hydrogen sources, pressure, continuous flow or batch conditions, reaction temperature, illuminated area, irradiance, spectral distribution, and reaction time. Whenever possible, catalytic performance should be reported on more than one normalization basis. In addition to NH_3_ production rates normalized by catalyst mass, studies would benefit from reporting area-normalized NH_3_ fluxes, metal-normalized or site-normalized productivity when meaningful, and photon- or energy-based metrics such as apparent quantum efficiency, wavelength-specific quantum efficiency, solar-to-ammonia efficiency, or overall energy efficiency when external heating is involved. Explicit reporting of the calculation basis and operating conditions for these quantities is essential if meaningful comparison across studies is to be achieved. Because NH_3_ synthesis rates are highly sensitive to the thermal state of the catalyst, the method used to determine temperature should always be specified explicitly, together with sensor position and any relevant calibration or correction procedure.^10–12,19,21,22^ Ammonia production should be accompanied by dark controls, catalyst-free controls, blank tests designed to exclude adventitious nitrogen sources and, ideally, isotopic validation when mechanistic claims are being made.^4,6^

At the mechanistic level, stronger standards are needed. In particular, claims that illumination alters the intrinsic catalytic pathway should not rely on activity enhancement alone. The minimum evidence for such an assignment should include, at minimum, a nominally matched-temperature light/dark comparison and one additional line of discrimination, such as a change in apparent activation energy, altered reaction orders, wavelength- or intensity-dependent behavior at controlled temperature, isotopic evidence, or *operando* spectroscopic support consistent with the proposed mechanism.^10–12,16,19,20^ If local temperature gradients are likely to be important, the uncertainty associated with temperature determination should be discussed explicitly rather than treated as a negligible experimental detail.^11,12,21–23^ Conversely, when illumination is inferred to act primarily through photocarrier participation, physical evidence of excited-state generation should be linked to chemical evidence that the catalytic pathway is modified in a way consistent with those excited states, rather than simply reported in parallel.

On this basis, a practical minimum-evidence hierarchy can be proposed. Photo-driven thermocatalysis can be assigned with relatively high confidence when illumination enhances NH_3_ synthesis but no convincing perturbation of the effective kinetic response is observed beyond heating or heat redistribution. Photo-assisted thermocatalysis requires stronger support, namely evidence that illumination modifies the effective kinetics of an otherwise thermally activated pathway under nominally matched thermal conditions. Photothermal co-catalysis should be reserved for systems in which thermochemical and photoinduced pathways both appear to contribute directly and cooperatively to the observed catalytic response, and in which that coupled behavior cannot be described adequately as either photo-assisted thermocatalysis or thermally assisted photocatalysis alone. Thermally assisted photocatalysis should be reserved for systems in which dark activity is negligible or strongly suppressed and the combined spectroscopic, kinetic, and optical evidence indicates that photoexcitation governs the key activation chemistry while heat mainly assists downstream processes. In all four cases, the strength of evidence should reflect not only the amount of data reported, but also how well alternative thermal explanations have been constrained.

More broadly, reporting practices should reflect the fact that mechanistic interpretation in photothermal catalysis is inherently reactor-sensitive. Reactor geometry, illumination profile, optical penetration, catalyst-bed thickness, and heat-transfer conditions can all influence the apparent balance between thermal and photoinduced effects.^10–12,21^ For this reason, studies that aim to make strong mechanistic claims should report enough reactor information to allow the reader to judge whether local heating, transport limitations, or spatially heterogeneous illumination may influence the conclusion. The goal is not to impose a rigid universal protocol on a rapidly developing field, but to ensure that mechanistic assignments are made with a level of transparency proportional to their strength.

Ultimately, the most useful standard for the field is not a single preferred metric, but a more disciplined correspondence between the strength of the mechanistic claim and the quality of the supporting evidence. This principle underlies the diagnostic framework proposed in this Review and provides a practical route for comparing future studies without forcing them into overly rigid categories. As photothermal ammonia synthesis continues to evolve, such reporting discipline will be essential for distinguishing genuine mechanistic advances from improvements that arise primarily from changes in thermal management, reactor design, or operating window.

## 7 Perspectives and Outlook

Photothermal ammonia synthesis has now reached a stage at which the central challenge is no longer simply to demonstrate light-assisted NH_3_ formation, but to determine with greater precision how illumination modifies catalytic function and under what conditions those effects become mechanistically significant. As discussed throughout this Review, the main limitation of the field is therefore no longer a lack of catalytic phenomena, but a lack of sufficiently consistent experimental discrimination to compare those phenomena on equal terms.^6,10–12^

A first priority for future work is the design of catalyst architectures in which light-responsive functionality overlaps as directly as possible with the kinetically relevant elementary step. This principle emerges repeatedly across the strongest cases discussed in this Review. In Ru-based systems, the most informative examples are those in which illumination acts at reducible or defect-rich interfaces that are already central to N_2_ activation or hydrogen management.^29–32^ In Fe-based systems, the clearest mechanistic effects appear when illumination perturbs structurally sensitive active environments involved in nitrogen activation, hydrogen coverage control, or spillover-mediated chemistry.^36,37,39,40^ Future catalyst design should therefore move beyond the simple combination of an active metal and a light absorber, and instead focus on interfaces where photoinduced charge redistribution, thermal activation, and chemically decisive bond activation are deliberately colocalized.

A second priority is the development of more reliable strategies for thermal discrimination under operating conditions. As emphasized throughout this Review, the strongest mechanistic uncertainty in photothermal ammonia synthesis remains the distinction between intrinsic light-induced perturbation and unresolved local heating.^10–12,16,19–23^ Future studies would benefit from combining multiple thermometric approaches with reactor-specific heat-transfer analysis, rather than relying on a single nominal catalyst temperature. In parallel, matched-temperature light/dark experiments should be complemented more routinely by kinetic observables such as reaction orders, apparent activation energies, isotope exchange, or optical control strategies that test whether the same catalytic response can be reproduced without direct catalyst photoexcitation.^6,10–12,16,19,20^

A third priority concerns operando mechanistic validation. If photothermal catalysis is to move beyond phenomenological rate enhancement, then future work must better connect physical evidence of excited-state generation with chemical evidence that the kinetically relevant pathway has been modified accordingly. In practical terms, this means that operando spectroscopy, isotope tracing, and kinetic analysis should be designed as mutually reinforcing tools rather than as isolated characterization add-ons. This need is already apparent in the strongest mechanistic studies discussed above, where temperature-normalized kinetics and interfacial evidence are most convincing when they converge on the same interpretation.^30,31,33,37,40^

Reactor design is likely to become equally important. Photothermal ammonia synthesis is intrinsically sensitive to photon penetration, catalyst-bed thickness, heat dissipation, and spatially heterogeneous illumination.^10–12,21–23^ This means that reactor geometry is not merely an engineering variable, but part of the mechanistic problem itself. Future advances may therefore come not only from improved catalysts, but also from reactor concepts that deliberately control the spatial relationship between light absorption, heat generation, and catalytic turnover.

From a practical design perspective, three priorities emerge. First, catalyst architectures should maximize the spatial overlap between light absorption, interfacial charge redistribution, and the kinetically relevant elementary step. Second, mechanistic studies should be designed from the outset to discriminate local heating from intrinsic light-induced perturbation, ideally by combining matched-temperature light/dark comparisons, multiscale thermometry, and at least one additional kinetic, isotopic, or operando spectroscopic discriminator. Third, reactor configurations should be treated as mechanistically relevant design variables: in some cases they should minimize thermal ambiguity to enable rigorous assignment, whereas in others they may be deliberately engineered to exploit beneficial thermal gradients while making that thermal contribution explicit.

Beyond these immediate priorities, progress in the field will depend on adopting a more disciplined correspondence between the strength of mechanistic claims and the quality of supporting evidence. This does not require a rigid universal protocol, nor does it imply that all future systems must converge toward a single preferred photothermal regime. Rather, it requires that claims of photo-driven thermocatalysis, photo-assisted thermocatalysis, photothermal co-catalysis, or thermally assisted photocatalysis be supported by evidence proportional to their interpretive ambition. In this sense, the most important advance for the field may be conceptual as much as catalytic: replacing overly broad claims of “photothermal synergy” with evidence-based assignments that make explicit both the likely dominant photothermal regime and the strength of evidence associated with that interpretation.

Ultimately, the future of photothermal ammonia synthesis will depend on whether catalyst design, mechanistic analysis, and reactor engineering can be brought into closer alignment. If that alignment is achieved, the field will be better positioned not only to improve NH_3_ productivity, but also to identify which light–heat interactions represent genuine mechanistic opportunities and which primarily reflect improved thermal management. That distinction will be essential for transforming photothermal ammonia synthesis from a promising collection of heterogeneous observations into a more predictive and designable catalytic discipline.
